# Supplementary material for: From Interconnection between Genes and Microenvironment to Novel Immunotherapeutic Approaches in Upper Gastro-Intestinal Cancers—A Multidisciplinary Perspective
Source: Cancers (Basel). 2020 Jul 29;12(8):2105. doi: 10.3390/cancers12082105 (PMC7465773; doi:10.3390/cancers12082105)
Supplement: Supplementary file 1 [file cancers-12-02105-s001.pdf]

Supplementary material

Oesohapgeal cancer.

The currently AJCC 8th edition staging of oesophageal, esophagogastric and gastric epithelial cancer includes three different staging groups: i) the clinical (cTNM) stage group, which considers depth of primary tumour invasion (T), regional lymph node involvement (N) and presence of metastases (M) (Suppl. Fig.1 and 2); ii) the pathologic (pTNM) stage group, which also includes hystologic grading; iii) the newly introduced post-neoadjuvant pathologic (ypTNM) stage group (Suppl. Fig.3), referred to patients who have undergone neoadjuvant therapy and pathologic review of the resection specimen, since tumor responsiveness to neoadjuvant therapy drives survival outcomes. Tumor cell grading is defined in Suppl. Tab. 3. The definition of the esophagogastric junction has been revised in the 8th edition such that cancers involving it with epicenters no more than 2 cm into the gastric cardia are staged as carcinomas of the oesophagus and those with more than 2-cm involvement of the gastric cardia are staged as stomach cancers. A new regional lymph node map has been developed to keep out lung lymph node stations included in the 7th edition of AJCC staging system. Regional lymph nodes include lower cervical paratracheal, upper/lower paratracheal, subcarinal, upper/middle/lower thoracic paraesophageal, pulmonary ligament, diaphragmatic, paracardial, left gastric, common hepatic, splenic, celiac and cervical periesophageal level VI/VII nodes. Moreover, ESCC is subdivided based on the location: i) X: Location unknown, ii) Upper: Cervical oesophagus to lower border of azygous vein, iii) Middle: Lower border of azygous vein to lower border of inferior pulmonary vein, iv) Lower: Lower border of inferior pulmonary vein to stomach, including gastroesophageal junction.

Supplementary Table 1. TNM staging for oesophageal cancer.

**T (primary tumor)**	
**Tx**	Tumor cannot be assessed
**T0**	No evidence of primary tumor
**Tis**	High grade dysplasia, defined as malignant cells confined to the epithelium by the basement membrane
**T1**	Tumor invades the lamina propria, [1]muscularis mucosae or submucosa<br>• **T1a:** Tumor invades the lamina propria or muscularis mucosae<br>• **T1b:** Tumor invades the submucosa
**T2**	Tumor invades the muscularis propria
**T3**	Tumor invades adventitia
**T4**	Tumor invades adjacent structures<br>• **T4a:** Tumor invades the pleura, pericardium, azygos vein, diaphragm or peritoneum<br>• **T4b:** Tumor invades other adjacent structures, such as the aorta, vertebral body or airway
**N (regional lymph nodes)**	
**Nx**	Regional lymph nodes cannot be assessed
**N0**	No regional lymph node metastasis
**N1**	Metastasis in 1 or 2 regional lymph nodes
**N2**	Metastasis in 3 - 6 regional lymph nodes
**N3**	Metastasis in 7+ regional lymph nodes
**M (distant metastases)**	
**M0**	No distant metastasis
**M1**	Distant metastasis

Supplementary Table 2. TNM stage grouping for oesophageal cancer.

Squamous cell clinical staging			
**0**	cTis	cN0	cM0
**I**	cT1	cN0 - 1	cM0
**II**	cT2	cN0 - 1	cM0
	cT3	cN0	cM0
**III**	cT3	cN1	cM0
	cT1 - 3	cN2	cM0
**IVA**	cT4	cN0 - 2	cM0
	any cT	cN3	cM0
**IVB**	any cT	any cN	cM1

Squamous cell pathological staging					
**0**	pTis	pN0	pM0	G N/A	any location
**IA**	pT1a	pN0	pM0	G1, GX	any location
**IB**	pT1a	pN0	pM0	G2 – 3	any location
	pT1b	pN0	pM0	G1 - 3, GX	any location
	pT2	pN0	pM0	G1	any location
**IIA**	pT2	pN0	pM0	G2 - 3, GX	any location
	pT3	pN0	pM0	any G	lower
	pT3	pN0	pM0	G1	upper / middle
**IIB**	pT3	pN0	pM0	G2 - 3	upper / middle
	pT3	pN0	pM0	GX	any location
	pT3	pN0	pM0	any G	location X
	pT1	pN1	pM0	any G	any location
**IIIA**	pT1	pN2	pM0	any G	any location
	pT2	pN1	pM0	any G	any location
**IIIB**	pT2	pN2	pM0	any G	any location
	pT3	pN1 - 2	pM0	any G	any location
	pT4a	pN0 - 1	pM0	any G	any location
**IVA**	pT4a	pN2	pM0	any G	any location
	pT4b	pN0 - 2	pM0	any G	any location
	any pT	pN3	pM0	any G	any location
**IVB**	any pT	any pN	pM1	any G	any location

Adenocarcinoma clinical staging			
**0**	cTis	cN0	cM0
**I**	cT1	cN0	cM0
**IIA**	cT1	cN1	cM0
**IIB**	cT2	cN0	cM0
**III**	cT2	cN1	cM0
	cT3	cN0 – 1	cM0
	cT4a	cN0 – 1	cM0
**IVA**	cT1 - 4a	cN2	cM0
	cT4b	cN0 – 2	cM0
	any cT	cN3	cM0
**IVB**	any cT	any cN	cM1

Adenocarcinoma pathological staging				
**0**	pTis	pN0	pM0	G N/A
**IA**	pT1a	pN0	pM0	G1, GX
**IB**	pT1a	pN0	pM0	G2
	pT1b	pN0	pM0	G1 - 2, GX
**IC**	pT1	pN0	pM0	G3
	pT2	pN0	pM0	G1 - 2
**IIA**	pT2	pN0	pM0	G3, GX
**IIB**	pT1	pN1	pM0	any G
	pT3	pN0	pM0	any G
**IIIA**	pT1	pN2	pM0	any G
	pT2	pN1	pM0	any G
**IIIB**	pT2	pN2	pM0	any G
	pT3	pN1 - 2	pM0	any G
	pT4a	pN0 - 1	pM0	any G
**IVA**	pT4a	pN2	pM0	any G
	pT4b	pN0 - 2	pM0	any G
	any pT	pN3	pM0	any G
**IVB**	any pT	any pN	pM1	any G

Supplementary Table 3. Post-neoadjuvant pathologic stage grouping.

Squamous cell/adenocarcinoma pathologic staging following neoadjuvant therapy			
**I**	ypT0 - 2	ypN0	ypM0
**II**	ypT3	ypN0	ypM0
**IIIA**	ypT0 - 2	ypN1	ypM0
**IIIB**	ypT3	ypN1	ypM0
	ypT0 - 3	ypN2	ypM0
	ypT4a	ypN0	ypM0
**IVA**	ypT4a	ypN1 - 2, X	ypM0
	ypT4b	ypN0 - 2	ypM0
	any ypT	ypN3	ypM0
**IVB**	any ypT	any ypN	ypM1

Supplementary Table 4. Tumor cell grading for oesophageal cancer.

	Adenocarcinoma	Squamous cell carcinoma
**GX**	Differentiation cannot be assessed	Differentiation cannot be assessed
**G1**	Well differentiated, with >95% of the tumour composed of well-formed glands	Well differentiated; prominent keratinization with pearl formation and a minor component of non-keratinizing basal-like cells, tumour cells arranged in sheets and mitotic counts low
**G2**	Moderately differentiated, with 50-95% of the tumor showing gland formation	Moderately differentiated with variable hystologic features ranging from parakeratotic to poorly keratinizing lesions and pearl formation generally absent
**G3**	Poorly differentiated, with tumors composed of nest and sheets of cells with <50% of the tumor demonstrating glandular formation	Poorly differentiated, consisting predominantly of basal like cells forming large and small nests with frequent central necrosis and with the nests consisting of sheets or pavement-like arrangements of tumor cells that are occasionally punctuated by small numbers of parakeratotic or keratinizing cells

Gastric cancer.

Supplementary Table 5. TNM staging for oesophageal cancer.

Regional lymph nodes include the greater curvature, greater omental, lesser curvature, lesser omental, right and left peracardial (cardioesophageal), suprapyloric, gastroduodenal, infrapyloric, gastroepiploic, left gastric artery, celiac artery, common hepatic artery, hepatoduodenal, portal, splenic artery, and splenic hilum nodes

T (primary tumor)	
**Tx**	No evidence of primary tumor
**T0**	No evidence of primary tumor
**Tis**	Carcinoma in situ: intraepithelial tumor without invasion of the lamina propria; high grade dysplasia
**T1**	Tumor invades the lamina propria, muscularis mucosae or submucosa<br>• **T1a**: Tumor invades the lamina propria or muscularis mucosae<br>**T1b**: Tumor invades the submucosa
**T2**	Tumor invades the muscularis propria
**T3**	Tumor penetrates the subserosal connective tissue without invasion of the visceral peritoneum or adjacent structures
**T4**	Tumor invades the serosa (visceral peritoneum) or adjacent structures<br>• **T4a**: Tumor invades the serosa (visceral peritoneum)<br>• **T4b**: Tumor invades adjacent structures/organs
**N (regional lymph nodes)**	
**Nx**	Regional lymph nodes cannot be assessed
**N0**	No regional lymph node metastasis
**N1**	Metastasis in one or two regional lymph nodes
**N2**	Metastasis in three to six regional lymph nodes
**N3**	Metastasis in seven or more regional lymph nodes<br>• **N3a**: Metastasis in seven to 15 regional lymph nodes<br>• **N3b**: Metastasis in 16 or more regional lymph nodes
**M (distant metastases)**	
**M0**	No distant metastasis
**M1**	Distant metastasis

Supplementary Table 6. Pathologic TNM staging (A) and grouping (B) for gastric cancer.

A-Pathologic staging			
	**T**	**N**	**M**
**0**	Tis	N0	M0
**IA**	T1	N0	M0
**IB**	T1	N1	M0
	T2	N0	M0
**IIA**	T1	N2	M0
	T2	N1	M0
	T3	N0	M0
**IIB**	T1	N3a	M0
	T2	N2	M0
	T3	N1	M0
	T4a	N0	M0
**IIIA**	T2	N3a	M0
	T3	N2	M0
	T4a	N1-2	M0
	T4b	N0	M0
**IIIB**	T1-2	N3b	M0
	T3-4a	N3a	M0
	T4b	N1-2	M0
**IIIC**	T3-4a	N3b	M0
	T4b	N3a-3b	M0
**IV**	any T	any N	M1

B-Clinical groups			
	**T**	**N**	**M**
**0**	Tis	N0	M0
**I**	T1-2	N0	M0
**IIA**	T1-2	N1-3	M0
**IIB**	T3-4a	N0	M0
**III**	T3-4a	N1-3	M0
**IVA**	T4b	N1-3	M0
**IVB**	any T	any N	M1

Supplementary Table 7. Pathologic staging following neoadjuvant therapy in gastric cancer.

Pathologic staging following neoadjuvant therapy			
**I**	T1-2	N0	M0
	T1	N1	M0
**II**	T3-4a	N0	M0
	T2-3	N1	M0
	T1-2	N2	M0
	T1	N3	M0
**III**	T4b	N0	M0
	T4a-4b	N1	M0
	T3, 4a, 4b	N2	M0
	T2, 3, 4a, 4b	N3	M0
**IV**	any T	any N	M1
